# Insensitivity to pain induced by a potent selective closed-state Nav1.7 inhibitor

**DOI:** 10.1038/srep39662

**Published:** 2017-01-03

**Authors:** M. Flinspach, Q. Xu, A. D. Piekarz, R. Fellows, R. Hagan, A. Gibbs, Y. Liu, R. A. Neff, J. Freedman, W. A. Eckert, M. Zhou, R. Bonesteel, M. W. Pennington, K. A. Eddinger, T. L. Yaksh, M. Hunter, R. V. Swanson, A. D. Wickenden

**Affiliations:** 1 Janssen R&D, L.L.C., 3210 Merryfield Row, San Diego, CA 92121, USA; 2 Peptides International, Louisville, KY 40299, USA; 3 University of California, San Diego, Department Anesthesiology and Pharmacology, 9500 Gilman Drive, La Jolla, CA 92093-0818, USA

## Abstract

Pain places a devastating burden on patients and society and current pain therapeutics exhibit limitations in efficacy, unwanted side effects and the potential for drug abuse and diversion. Although genetic evidence has clearly demonstrated that the voltage-gated sodium channel, Nav1.7, is critical to pain sensation in mammals, pharmacological inhibitors of Nav1.7 have not yet fully recapitulated the dramatic analgesia observed in Nav1.7-null subjects. Using the tarantula venom-peptide ProTX-II as a scaffold, we engineered a library of over 1500 venom-derived peptides and identified JNJ63955918 as a potent, highly selective, closed-state Nav1.7 blocking peptide. Here we show that JNJ63955918 induces a pharmacological insensitivity to pain that closely recapitulates key features of the Nav1.7-null phenotype seen in mice and humans. Our findings demonstrate that a high degree of selectivity, coupled with a closed-state dependent mechanism of action is required for strong efficacy and indicate that peptides such as JNJ63955918 and other suitably optimized Nav1.7 inhibitors may represent viable non-opioid alternatives for the pharmacological treatment of severe pain.

Pain presents a major societal problem and current pain therapeutics exhibit limited efficacy, unwanted side effects and the potential for drug abuse and diversion. Two data sets have strongly implicated in humans, a pivotal role of Nav1.7 (also named as PN1, SCN9A or hNE) in nociceptive processing. First, homozygous or compound heterozygous loss-of-function mutations of Nav1.7 in humans lead to complete insensitivity to pain subsequent to high threshold stimuli, tissue injury and inflammation^1^,^2^,^3^. Second, gain-of-function mutations of Nav1.7 have been linked to primary erythromelalgia (PE) and paroxysmal extreme pain disorder (PEPD), autosomal dominant disorders characterized by episodic burning pain and redness of the extremities and other peripheral systems^4^,^5^. These phenotypic characteristics are preserved in animal models. Thus, global Nav1.7 knockout mice are i) completely insensitive to acute mechanical, thermal, and chemical noxious stimuli, ii) show no nocifensive behaviors resulting from peripheral injection of sodium channel activators, and iii) do not develop hyperalgesia following adjuvant-induced inflammation^6^. Likewise, deleting Nav1.7 in both sensory and sympathetic neurons abolishes mechanical, thermal and neuropathic pain^7^. Conditional Nav1.7 knock-out in adult mice results in a similar phenotype, suggesting that the profound loss of pain sensation is not due to a neurodevelopmental deficit^8^. Mechanistically, this phenotype is consistent with the finding that Nav1.7 is prominently expressed in small diameter, non-myelinated fibers (nociceptive neurons), where it is thought to amplify small sub-threshold depolarizations to regulate firing^9^.

The strong genetic evidence that Nav1.7 is critical to pain sensation in man and rodents suggests that pharmacological inhibition of Nav1.7 function should provide powerful analgesia. However, although several selective Nav1.7 inhibitors have been described in the literature^10^,^11^,^12^, none have fully recapitulated the dramatic analgesia observed in Nav1.7-null subjects^11^,^12^ and clinical progress has been slow^13^,^14^. While the absence of efficacy has discouraged many in the field, and led some to question the drugabililty of Nav1.7, one possible explanation is that the pharmacological tools utilized provided sub-optimal block of Nav1.7. Indeed, all so-called selective small molecule Nav1.7 blockers described to date are only partially selective^12^,^15^,^16^ and inhibition of sodium channel isoforms other than Nav1.7 may preclude evaluation of maximally effective Nav1.7 blocking doses *in-vivo*. Furthermore, all small molecule sodium channel blockers identified to date exhibit a mechanism of action involving state-dependence^15^,^16^,^17^, preferentially binding to and stabilizing the inactivated state, thereby reducing the number of channels available to open during subsequent depolarizations. However, since it is difficult to predict the extent of access to the inactivated state *in-vivo*, it is possible that channel block by state-dependent small molecules will only be partial under (patho)physiological conditions. Therefore, pharmacological agents with a high degree of Nav1.7 selectivity and a mechanism of action that allows binding to all physiological channel states may be required for optimal efficacy.

Spider venoms are a rich source of potent sodium channel modulating peptides. Protoxin-II (ProTX-II; β/ω-theraphotoxin-Tp2a), a 30 amino acid family 3 inhibitor cystine knot peptide from *Thrixopelma pruriens,* (green velvet tarantula), is a potent (IC_50_ < 1 nM) and selective (>30x) Nav1.7 blocker^11^,^18^ that exhibits a mechanism of action that has been highly optimized through venom evolution to powerfully inhibit nervous system ion channels under *in-vivo* conditions and thereby maximize efficacy. Although previous studies have suggested limited efficacy of ProTX-II in rodent pain models^11^, we now show that this is likely due to a small therapeutic window and that efficacy can indeed be demonstrated in a narrow dose range. Using ProTX-II as a scaffold, we engineered a Nav1.7 blocking peptide, JNJ63955918, with improved Nav1.7 selectivity and *in-vivo* tolerability. Here we show that JNJ63955918 induces a pharmacological insensitivity to pain that fully recapitulates the Nav1.7-null phenotype.

## Results

### *In-vivo* efficacy of ProTX-II

Previous studies have suggested that ProTX-II may not penetrate the peripheral nerve sheath very effectively^19^. Therefore, we initially focused on IT administration of ProTX-II to ensure the peptide had access to target sites within the dorsal root and pre-synaptic sensory nerve endings within the spinal cord. Previous reports on the efficacy of ProTX-II by the IT route of administration are mixed^11^,^20^. We therefore re-evaluated the analgesic effects of intrathecal ProTX-II in rat models of thermal and chemical nociception.

**Figure 1:**
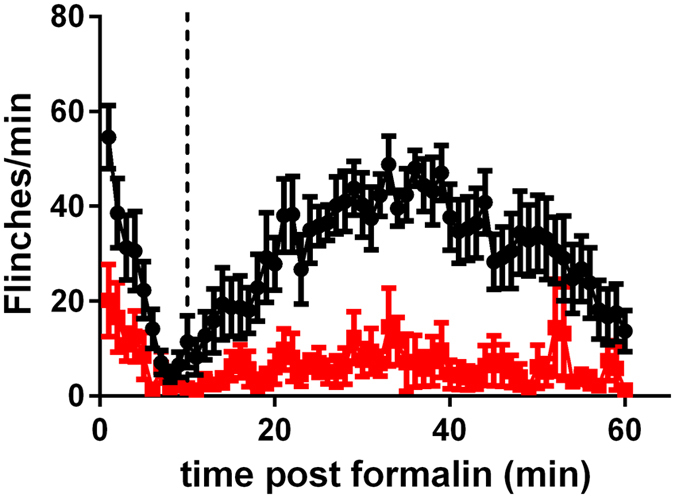
Effects of ProTX-II on formalin-induced flinching in rats. Effect of vehicle (●) or ProTX-II (2 μg/ 10 μl per rat I.T., 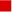) on formalin-induced flinching. The vertical dotted line separates phase I (0–10 min) from phase II (11–60 min).

In dose finding studies, the maximum tolerated IT dose of ProTX-II was 2 μg/10 μl. Higher doses produced dose-related motor abnormalities that progressed from transient rear weakness, to paralysis of both the hind and forelimbs, slowing of respiration and death. In the Hargreaves test, animals dosed with either 2 μg/10 μl or 1.6 μg/10 μl (but not 0.8μg/10 μl) ProTX-II exhibited elevated thermal latencies compared to their baselines starting at 30 min and lasting through 4 h. By 24 h, latencies had returned to baseline values. Based on these observations, a dose of 2 μg/10 μl ProTX-II was evaluated in a rat formalin study. IT injections of 2 μg/10 μl ProTX-II produced a highly significant reduction in phase I and phase II flinching compared to vehicle treated rats (Fig. 1) without any severe effect on motor function (Supplementary Table 1). Abrasions and scabs to the face, neck, and shoulders were observed in some ProTX-II treated animals.

These findings show that ProTX-II does indeed exert a strong analgesic effect following IT injection. Dose finding studies also indicated that ProTX-II has a steep dose-response relationship and exerts profound motor effects at doses just above the efficacious analgesic dose, presumably as a result of inhibition of sodium channel isoforms present on motor neurons e.g., Nav1.1 and Nav1.6. In an effort to capitalize on our observation of strong analgesic efficacy but limited therapeutic window of ProTX-II, we next sought to improve the selectivity for Nav1.7 over other isoforms through peptide engineering on the ProTX-II scaffold.

### Engineering strategy leading to discovery of JNJ63955918

**Figure 2:**
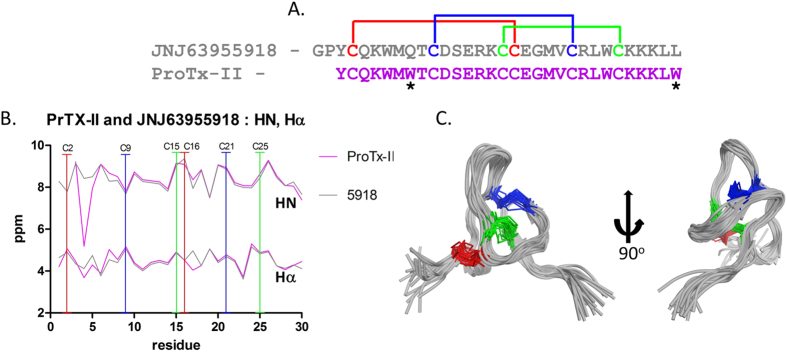
Sequence and solution structure of JNJ63955918. (**A**) The sequence of JNJ63955918 and ProTX-II highlighting cysteine residues with disulfide connectivity, asterisks indicate residue differences. (**B**) Backbone chemical shifts (HN, Hα) of ProTx-II and JNJ63955918 (see Supplementary Table 4 for chemical shifts). (**C**) Backbone superimposition of the ensemble (20 structures) of lowest energy conformers of JNJ63955918 (pdb accession number 5TCZ, BMRB accession number 30181). Disulfide CYS pairings are shown as blue, red, and green bonds.).

We initially performed single position amino acid scanning mutagenesis on ProTX-II as part of a broad effort to identify ProTX-II analogs with improved selectivity. Each of the 24 non-cysteine positions in ProTX-II were systematically substituted with every coded amino acid except for methionine and cysteine. In a second round of engineering, single position substitutions that showed improved selectivity or improved recombinant peptide yield were evaluated combinatorially. In total, we generated a unique library of over 1500 ProTX-II-related peptides^21^,^22^. From the initial SAR exploration we identified W30L as a substitution that significantly improved the sodium channel selectivity in favor of Nav1.7 (pIC_50_ values for ProTX-II W30L were 8.3, 6.3 and 5.4 for Nav1.7, Nav1.6 and Nav1.4, respectively). Further improvements in selectivity were observed when W30L was combined with a second substitution, W7Q that improved refolding efficiency (as evidenced by the overall yield from crude in solid-phase peptide synthesis). This paper characterizes the pharmacology of GP-ProTX-II W7QW30L or JNJ63955918 (Fig. 2A).

### NMR structure of JNJ63955918

NMR was used to determine the solution structure of JNJ63955918 (Fig. 2). The data indicates JNJ63955918 has a very similar structure to the parent ProTX-II (Fig. 2B) and other related spider venom peptides^23^. These peptides adopt a condensed inhibitor cystine knot (ICK) fold stabilized by three conserved disulfide bonds (Fig. 2C). For ProTX-II and JNJ63955918 the backbone proton chemical shifts, HN and H-α, differ little over the length of the backbones (Fig. 2B). This, in addition to NOE data, suggests that the surrounding environments between equivalent residues in the peptides are not significantly different and that the global fold is the same (Supplementary Figure 2). The largest difference in backbone shifts occurs between K4 and W5, due to a strong ring current anisotropy, where the presence (ProTx-II), or absence (JNJ63955918), of a neighboring indole ring from W7 is apparent.

### Improved selectivity of JNJ63955918

**Figure 3:**
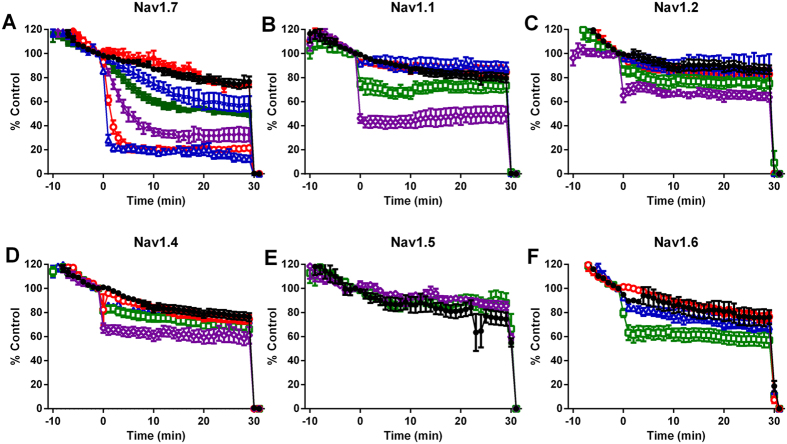
Concentration dependent inhibition of human Nav1 channels by JNJ63955918 measured in QPatch. Control (●); 1 nM (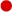); 3 nM (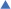); 10 nM (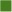); 30 nM (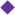); 100 nM (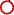); 300 nM (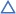); 1 μM (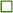); 3 μM (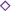) JNJ63955918. Positive control agents (TTX or lidocaine) were added at 30 min.

**Figure 4:**
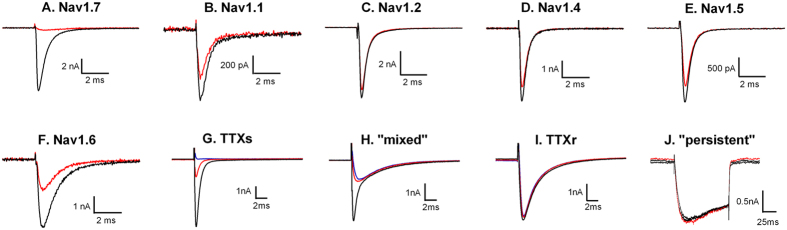
Representative traces to illustrate inhibition of human Nav1 channels by JNJ63955918 measured by manual patch clamp. Black traces are control, red traces are after addition of JNJ63955918 at either 1 μM (**A,C,D,F**), 3 μM (**B**) or 10 μM (**E**). (**G–J**) show representative TTX-sensitive (**G**), mixed (**H**), TTX-resistant (**I**) and persistent (**J**) sodium currents recorded from small and medium diameter rat DRG neurons. Black lines are control responses, red lines are currents in the presence of 300 nM JNJ63955918 and blue lines are currents recorded in the presence of 1 μM TTX.

**Figure 5:**
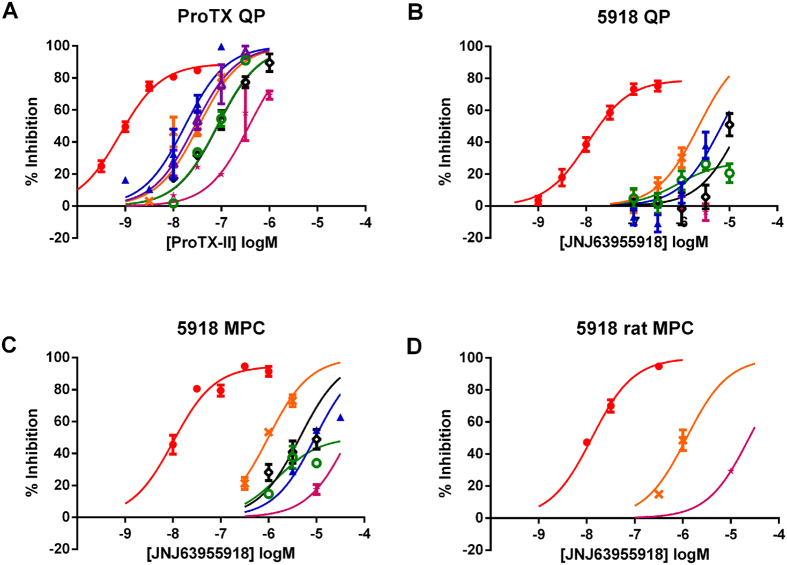
Human and rat Nav1.x pharmacology of ProTX-II and JNJ63955918. Concentration-dependent inhibition of Nav1.7 (red), Nav1.1 (blue), Nav1.2 (green), Nav1.3 (purple), Nav1.4 (black), Nav1.5 (pink) and Nav1.6 (orange) by ProTX-II (**A**) or JNJ63955918 (**B–D**). Data shown in panels A and B were generated using automated patch clamp. Data shown in (**C** and **D**) were generated by manual patch clamp. Data were generated using either human (**A–C**) or rat (**D**) sodium channel isoforms.

We have previously reported the validation of an automated patch clamp assay with sensitivity comparable to manual patch clamp^24^. In this QPatch assay, ProTX-II was a potent inhibitor of Nav1.7 (pIC_50_ = 9.1) with selectivity over other sodium channel isoforms ranging from 19.8-fold (vs Nav1.1) to 497.6-fold (vs Nav1.5, Table 1). JNJ63955918 was a potent and selective inhibitor of human Nav1.7 in automated and manual patch clamp assays (Figs 3, 4 and 5). JNJ63955918 was similarly selective for rat Nav1.7 over rat Nav1.6 and rat Nav1.5 (Fig. 5D). pIC_50_ values are shown in Table 1. Importantly, although less potent, JNJ63955918 exhibited improved selectivity for Nav1.7 over other Nav1.x isoforms compared to ProTX-II (Table 1, Fig. 5). The observed improvements in selectivity and refolding resulted from the W7Q, W30L mutations rather than the additional two residues (G,P) on the N-terminus (pIC_50_ values for ProTX-II W7Q, W30L lacking the extra GP residues were 8.0, 5.7 and 5.4 for Nav1.7, Nav1.6 and Nav1.4, respectively). The effects of JNJ63955918 were fully reversible. Reversal of Nav1.7 inhibition was slow at hyperpolarized membrane potentials but greatly accelerated by holding at 0 mV during washout. Consistent with lower affinity, washout was rapid even at hyperpolarized membrane potentials for other Nav1.x isoforms. JNJ63955918 had no agonist or antagonist activity at mu, delta or kappa opioid receptors (pIC_50_ or pEC_50_ < 5.6).

### Rat dorsal root ganglion studies

Sodium currents in small to medium diameter DRG cells were classified as either predominantly TTX-sensitive (5/12; ~100% block by 1 μM TTX), predominantly TTX-resistant (4/12, <10% block by 1 μM TTX), mixed (3/12, 29–68% block by 1 μM TTX) or persistent (slowly activating, slowly inactivating TTX-resistant). JNJ63955918 (300 nM) had no effect on TTX-resistant (3 ± 3% inhibition, n = 4; Fig. 4I) or persistent (−4 ± 0.9% inhibition, n = 3; Fig. 4J) currents. In contrast, 300 nM JNJ63955918 inhibited 78 ± 3% of the TTX-sensitive current in TTX-sensitive and mixed cells (Fig. 4G,H). These findings suggest that Nav1.7 underlies the majority of the TTX-sensitive current in small and medium diameter rat DRG cells and that JNJ63955918 exhibits substantial selectivity for Nav1.7 over Nav1.8 and Nav1.9. These findings are consistent with previous estimates of the contribution of Nav1.7 in mouse (83%) and human (75.5%) DRGs^15^,^25^.

### Mechanism of action of JNJ63955918

**Figure 6:**
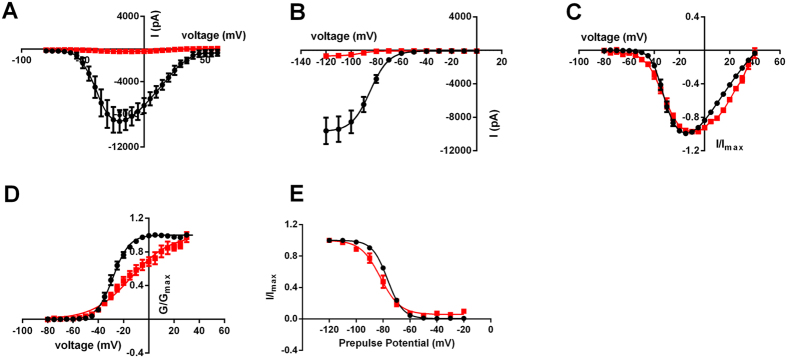
Closed-state block of hNav1.7 by JNJ63855918. (**A**) Average current-voltage relationship for hNav1.7 before (black) or after (red) addition of 300 nM JNJ63955918 (**B**) Average steady-state inactivation curves for hNav1.7 before (black) or after (red) addition of 300 nM JNJ63955918 (**C**) Normalized current-voltage curves measured in time-matched control cells (black) and cells exposed to 300 nM JNJ63955918 (red) (**D**) Normalized activation curves derived from the data shown in (C). (**E**) Normalized steady-state inactivation curves measured in time-matched control cells (black) and cells exposed to 300 nM JNJ63955918 (red).

In manual patch clamp studies JNJ63955918 potently inhibited Nav1.7 currents elicited from a holding potential of −120 mV, suggesting that the peptide binds effectively to closed or resting channels (Figs 4A, 5C,D and 6A,B). Furthermore, the close agreement between QPatch and manual patch pIC_50_ values (Table 1 and Fig. 5B,C) despite the differences in holding potential (V_½_ inactivation for QP and −120 mV for MPC) between these two assays suggests that JNJ63955918 is largely voltage-independent over a range of physiological voltages. In time-matched experiments the voltage for half activation was significantly shifted from −28.2 ± 1.5 mV (n = 6) under control conditions to −12.7 ± 3.5 mV (n = 7) in the presence of 300 nM JNJ63955918 (Fig. 6C,D). Slope values were also significantly shifted from 6.2 ± 0.3 under control conditions to 14.9 ± 1.3 in the presence of 300 nM JNJ63955918, possibly indicating a degree of channel unblock at depolarized potentials. JNJ63955918 also modestly shifted the voltage for half inactivation from −76.7 ± 0.8 mV under control conditions to −82.7 ± 2.2 mV in the presence of 300 nM JNJ63955918 (Fig. 6E). Slopes were similar under control conditions and in the presence of JNJ63955918 (5.5 ± 0.1 and 6.2 ± 0.5, respectively).

### *In vivo* intrathecal dose range finding for JNJ63955918

In preliminary studies, rats were carefully assessed for deficits in placing/stepping, muscle strength/flaccidity, righting, body symmetry/posture, symmetric ambulation/limp, startle response and pinna reflex following intrathecal dosing of JNJ63955918. Intrathecal doses up to 5 μg/10 μl were well tolerated with no detectable abnormal behaviors. Mild, transient (<1 h), fully reversible muscle weakness was observed in approximately 50% of rats dosed intrathecally with 7.5 μg/10 μl of JNJ63955918. Scabbing on face/neck/shoulders was observed in some rats. Dose related motor impairment became more obvious and the duration of the effects was longer at doses above 7.5 μg/10 μl.

### Intrathecal bolus administration

**Figure 7:**
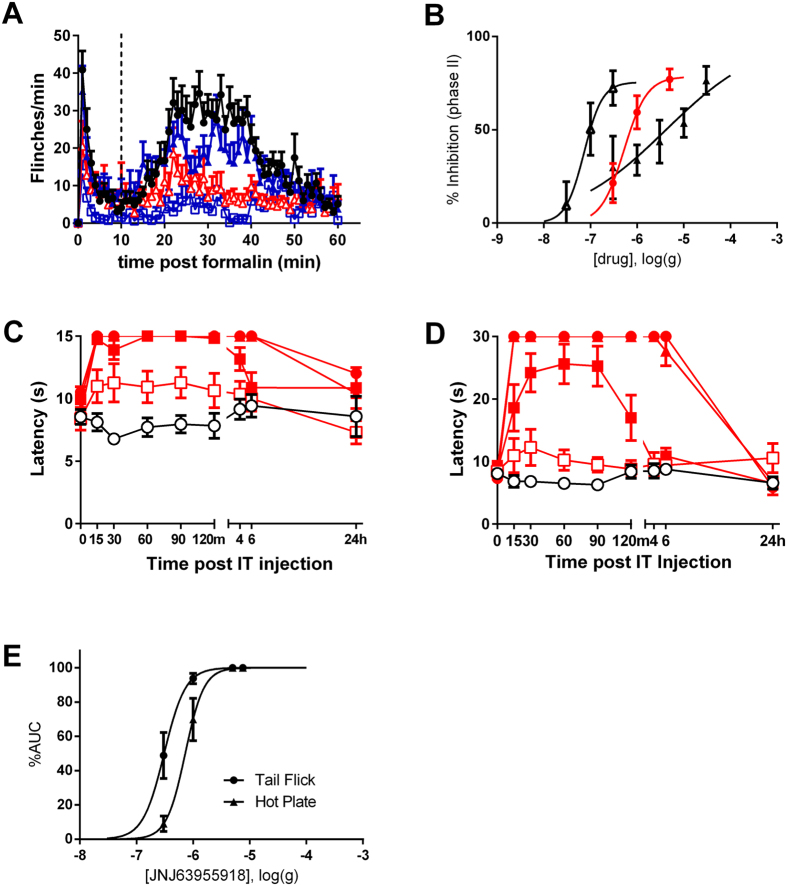
Effects of JNJ63955918, morphine and ziconotide in rat models of acute chemical and thermal pain. (**A**) Effect of vehicle (●) or 0.3 μg (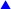), 1 μg (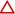) or 5 μg (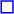) JNJ63955918 on formalin-induced flinching. The vertical dotted line separates phase I (0–10 min) from phase II (11–60 min). (**B**) Comparison of JNJ63955918 (red circles), ziconotide (open triangles) and morphine (closed triangles) in the formalin model. (**C**) Effect of intrathecal vehicle (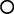) or 0.3 μg (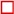), 1 μg (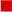), 5 μg (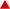) or 7.5 μg (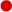) JNJ63955918 on thermally-induced tail flick. Latencies were significantly elevated between 15 and 120 min following 1 μg JNJ63955918 and between 15 and 360 min after 5 μg and 7.5 μg JNJ63955918 (2-way ANOVA with Bonferroni multiple comparisons vs baseline for each group). (**D**) Effect of intrathecal vehicle (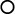) or 0.3 μg (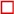), 1 μg (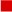), 5 μg (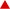) or 7.5 μg (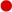) JNJ63955918 on escape latency in the hotplate assay. Latencies were significantly elevated between 15 and 120 min following 1 μg JNJ63955918 and between 15 and 360 min after 5 μg and 7.5 μg JNJ63955918 (2-way ANOVA with Bonferroni multiple comparisons vs baseline for each group). (**E**) Dose-response curves for JNJ63955918 in the rat tail flick and hotplate assays. Data are represented as mean ± s.e.m, n = 6–10 animals/group.

JNJ63955918 exhibited strong efficacy against chemical (Fig. 7A,B) and thermal (Fig. 7C,D,E) pain in rats following bolus intrathecal administration. pED_50_ values are presented in Table 2. The duration of the analgesia was dose- and model-dependent, with a single dose of 5 μg/10 μl JNJ63955918 providing almost complete analgesia for approximately 6 h post-dose in the hotplate and tail flick assays. The intrathecal efficacy of JNJ63955918 in the rat formalin model was replicated in a fully blinded, independent study in which JNJ63955918 significantly inhibited phase II formalin flinching at 0.5 μg/10 μl and 2.5 μg/10 μl. In this repeat study, excessive scratching behavior leading to upper body skin abrasion was observed in 2 of 4 animals dosed with 2.5 μg/10 μl JNJ63955918 and 1 of 4 animals dosed with 0.5 μg/10 μl JNJ63955918. Abrasions were observed on the forelimb, mandible, pinna, and head. No other untoward behaviors were observed (Supplementary Table 2). JNJ63955918 was equi-efficacious with intrathecal morphine and ziconotide against phase II formalin flinching in the rat (Fig. 7B and Table 2). Intrathecal Ziconotide produced whole body shakes, increased muscle tone and “serpentine” tail movement at all effective doses, as previously noted^26^.

### 14 day continuous infusion

**Figure 8:**
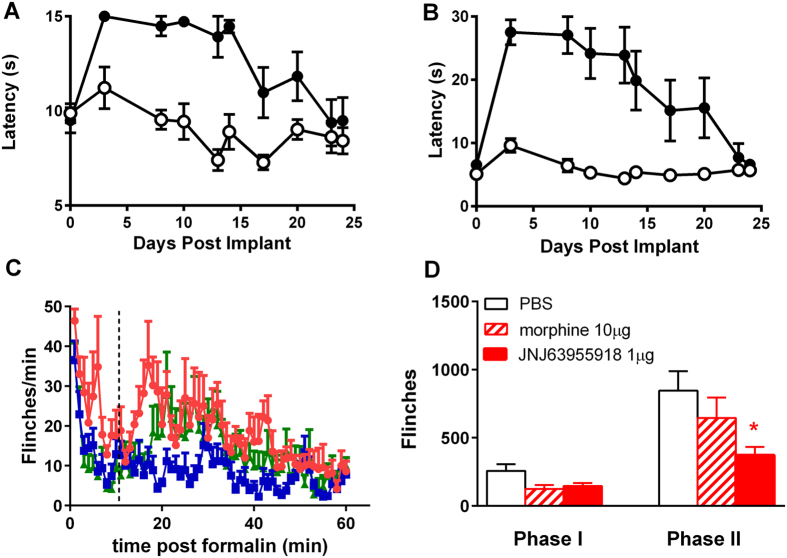
Lack of tolerance to JNJ63955918 and efficacy in morphine tolerant rats. Effect of 14 d intrathecal infusion of vehicle(○) or 0.5 μg/h JNJ63955918 (●) in the rat tail flick (**A**) and hotplate (**B**) models. Latencies were significantly elevated between day 3 and day 14 in the tail flick assay and between day 3 and day 20 in the hotplate assay (2-way ANOVA with Bonferroni multiple comparisons vs baseline for each group). (**C**) Effect of intrathecal vehicle (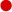), 1 μg JNJ63955918 (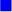) or 10 μg morphine (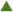) on formalin-induced flinching in morphine-tolerant rats. (**D**) Average effects of intrathecal vehicle, 1 μg JNJ63955918 or 10 μg morphine on phase I and phase II responses in the formalin model (*denotes P < 0.05, 1-way ANOVA). Phase I = 0~10 min and Phase II = 11~60 min. Data are represented as mean ± s.e.m, n = 5-8 rats/group.

JNJ63955918 (0.5 μg/h) was administered by continuous intrathecal infusion for ~14 d (actual lifetime of osmotic mini-pumps is variable), followed by ~7 d recovery. Efficacy was assessed in the hotplate and tail flick tests at various time points before pump implantation, during infusion and after discharge of the pumps. The findings are summarized in Fig. 8. JNJ63955918 (0.5 μg/h) exhibited significant efficacy that was maintained for 14 d in both tail flick (8A) and hotplate (8B) assays. Response latencies returned to baseline by day 25 (by which time all pumps should have been completely discharged). No motor or other behavioral abnormalities were observed over the course of the experiment. Drug- and vehicle-treated rats gained weight at the same rate over the course of the experiment (Supplementary Figure 3). Some scabbing on the face, shoulders, hind limbs and tail was noted in JNJ63955918 treated animals.

### Efficacy in morphine tolerant rats

In rats made tolerant to intrathecal morphine (by continuous infusion of 15 μg/h morphine for 7 d), a subsequent bolus injection of morphine (10 μg/10 μl) that was previously analgesic in morphine-naïve rats (Fig. 7B) did not significantly reduce phase II formalin flinching (Fig. 8C,D). In previous work we have shown that intrathecal morphine infusion results in a significant block of phase 2 on day 2, but as shown here loses its efficacy by day 7^27^. In contrast, JNJ63955918 (1 μg/10 μl) in morphine-tolerant rats at day 7 significantly reduced flinching to a similar degree to that observed in naïve rats (Figs 7B and 8C,D).

### Perineural Administration

**Figure 9:**
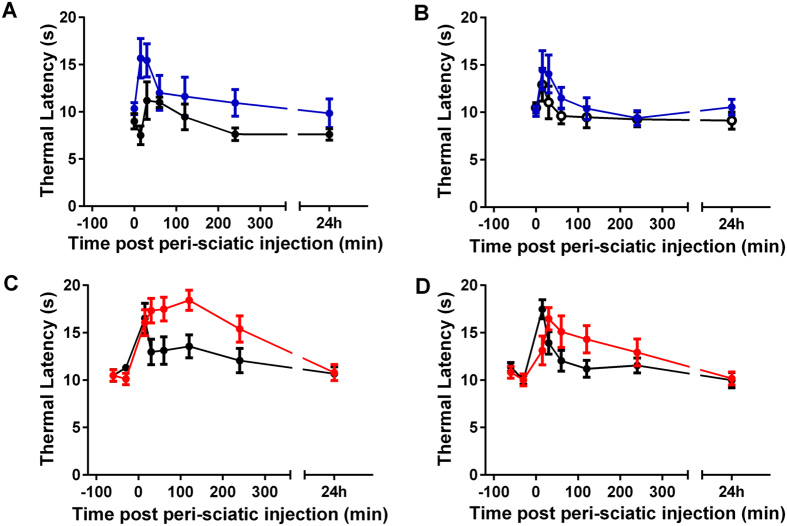
Effect of peri-sciatic ProTX-II and JNJ63955918 on thermally-induced pain in rats. Effect of peri-sciatic vehicle (●), 0.25 mg/rat ProTX-II (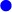) (**A,B**) or 1.4 mg/rat JNJ63955918 (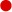) (**C,D**) on thermal withdrawal latencies of ipsilateral (**A,C**) and contralateral limbs (**B,D**). Comparison with a 2-way ANOVA with Bonferroni multiple comparisons across time and treatment revealed a statistically significant increase at 15 and 30 min for ProTX-II compared to vehicle, and at 15–240 min for JNJ63955918 compared to vehicle in the ipsilateral limb. Contralateral latencies were statistically increased from 30 min through 2 h by JNJ63955918 only.

Given the notable efficacy of ProTX-II and JNJ63955918 when administered locally to the spinal cord, we were interested to determine if these compounds were also efficacious by local administration at other sites along the sensory nerve. Peri-sciatic ProTX-II was well tolerated at the highest dose tested (250 μg/100 μl). Thermal latencies in the Hargreaves test were statistically elevated at 15 and 30 min in ProTX-II-treated animals compared to vehicle-treated animals (Fig. 9A). No statistically significant effects of ProTX-II were observed in the contralateral limb (Fig. 9B). No effect upon ambulation was noted for any animal during the course of the experiment.

In preliminary dose range finding studies peri-sciatic JNJ63955918 was well tolerated in rats up to the highest dose tested (2.5 mg/100 μl). At this dose, thermal latencies in the Hargreaves test were maximally elevated on both ipsilateral and contralateral paws for up to 2 h after injection in the absence of any gross motor deficits. Latencies returned to normal by 24 h post-dose. Peri-sciatic injection of 1.4 mg/100 μl and 0.8 mg/100 μl JNJ63955918 elevated thermal latencies primarily on the ipsilateral side, with no untoward effects. Based on these observations, the analgesic effects of 1.4 mg/100 μl JNJ63955918 was evaluated in additional animals following peri-sciatic dosing in the rat Hargreaves test. JNJ63955918 (1.4 mg/100 μl) produced a significant elevation of the thermal threshold, primarily in the ipsi-lateral limb (Fig. 9C). The effect lasted for 4 h and thresholds returned to normal by 24 h. A lesser effect of shorter duration was noted in the contra-lateral limb (Fig. 9D). Scratching was observed in some animals but no other behavioral abnormalities were noted (Supplementary Table 3).

## Discussion

In contrast to an earlier report^11^, our studies show that ProTX-II exerts anti-nociceptive effects in rat tests of thermal and chemical nociception after intrathecal and perineural delivery. However, ProTX-II has a very steep dose-response curve and has analgesic utility absent motor effects only over a very narrow dose range. Our studies also show that ProTX-II exerts profound motor effects at doses just above the efficacious dose range, presumably as a result of inhibition of other sodium channel isoforms present on motor neurons, such as Nav1.1 and Nav1.6, precluding further exploration of the dose-response relationship for analgesic activity. Through systematic mutagenesis of the ProTX-II scaffold, we identified JNJ63955918 (GP-ProTX-II W7Q, W30L) as a potent, selective closed-state Nav1.7 blocker. Importantly, JNJ63955918 exhibited improved Nav1.1, 1.2, 1.6 selectivity compared to ProTX-II. Based on the selectivity data for the individual mutations alone on wild-type ProTX-II we surmise that in the context of the double mutant, the W30L substitution is the source of the increase in selectivity for Nav1.7. It has been shown previously the N-methylation of a C-terminal amide of ProTX-II (ProTX-II-NH-Me) at W30 increases selectivity for Nav1.7 over Nav1.2^28^. Given the flexibility we observe in the C-terminus of JNJ63955918 by NMR, it is possible that a portion of the aliphatic side chain of the leucine mutant occupies a similar position within the voltage sensor as the aliphatic methyl group in ProTX-II-NH-Me, and therefore has a similar mechanism of selectivity. The potency difference between ProTX-II-NH-Me and JNJ63955918 suggests that the binding contacts may be similar but not identical. W7 has recently been reported to be an important component of a lipid interaction surface that includes residues K4, W5, M6 and W7 along with supplementary contributions from Y1, W24 and K27^29^. Based on association/dissociation rates, parts of this face of ProTX-II were deemed likely to be important for membrane partitioning but not for direct interaction with the channel voltage sensor^30^. The decrease in overall Nav1.7 potency that we observe in W7Q and the W7Q/W30L double mutant is consistent with other reports of mutations to this face and is possibly due to a decreased rate of membrane partitioning. However, as ProTX-II is notable for its synthetic intractability this potency decrease was counterbalanced by significant improvements in peptide manufacturability since it substantially increased the overall proportion of the correctly folded disulfide bonded isomer during solid-phase peptide synthesis and refolding.

Our mechanism of action studies show that JNJ63955918 binds effectively to the closed state to inhibit activation of Nav1.7 and experiments using different holding potentials demonstrate that inhibition is essentially voltage-insensitive over a physiological range. This mechanism contrasts greatly to the mechanism of small molecule sodium channel blockers such as acylsulfonamides^15^,^16^,^31^,^32^, local anesthetics, antiarrhythmic and anticonvulsant drugs^33^, that bind preferentially to the inactivated state. The voltage-dependent activation properties of the residual current in the presence of a saturating concentration of JNJ63955918 are modestly shifted and the slope is increased compared with control currents, suggesting a degree of unblock at relatively depolarized potentials. Reduced binding at depolarized potentials is also indicated by the accelerated recovery from block after holding at depolarized potentials during washout. Similar observations have been previously reported for ProTX-II^11^,^30^,^34^, indicating that JNJ63955918 retains a similar evolutionarily optimized molecular mechanism of action as the parent peptide, binding to, and immobilizing voltage-sensors and preventing channel activation under physiological conditions^34^,^35^.

JNJ63955918 was strongly active in several tests of acute, nociceptive pain following intrathecal administration. In each test close to complete suppression of pain behaviors was observed. The insensitivity to pain in the absence of other obvious sensory or motor deficits following administration of JNJ63955918 closely resembles the remarkable phenotype in Nav1.7-null mice and humans^6^,^7^. Lesions to the face and shoulders were often noticed in rats treated with JNJ63955918 and ProTX-II. Although not directly observed or quantified during our behavioral studies, we hypothesize that JNJ63955918 and ProTX-II induce scratching behavior or excessive/abnormal grooming. As charged peptides, it is possible that JNJ63955918 and ProTX-II non-specifically induce itch through mast cell degranulation and histamine release. Indeed, ziconotide, a similarly charged peptide has been shown to induce histamine release and associated hemodynamic changes following high intravenous doses in rats^36^. However in the current study, intrathecally administered ziconotide did not induce scratching behavior, suggesting other mechanisms could be involved. Interestingly, the appearance of facial lesions has been noted in a preliminary description of conditional Nav1.7 knock-out mice^37^, suggesting that the phenomenon is Nav1.7-mediated rather than compound-related. It is possible that loss of Nav1.7 and the associated inhibition of nociceptive input to the spinal cord lead to abnormal sensory processing and enhanced itch. A similar mechanism has been proposed to underlie in part the itch induced by morphine^38^. Alternatively, lack of pain sensation may lead to excessive grooming in rats. Further studies are needed to understand the role of Nav1.7 in itch sensation.

To our knowledge, this is the first time pharmacological inhibition of Nav1.7 has been shown to recapitulate two key hallmarks of Nav1.7 knock-out in mice, i.e., insensitivity to pain and facial lesions/scratching. A third feature of Nav1.7 KO in humans and mice is anosmia^6^,^39^. Olfactory function was not measured in our studies, thus it is not clear whether pharmacological block of Nav1.7 with intrathecal JNJ63955918 in rats can also recapitulate this feature of Nav1.7 KO. Although two recent studies have also reported a profound loss of pain sensation following administration of either a Nav1.7 blocking peptide from Centipede venom^40^, or an anti-Nav1.7 monoclonal antibody^41^, neither of these molecules were reported to induce facial lesions or anosmia. The inability to fully recapitulate the features of Nav1.7 KO perhaps indicates a different level of Nav1.7 blockade with these agents.

A recent study has suggested that endogenous opioids may contribute to insensitivity to pain in Nav1.7-null humans and mice^42^. Since tolerance did not develop to JNJ63955918 over 14 days of constant intrathecal infusion and since JNJ63955818 retained full efficacy in rats made tolerant to morphine, it appears that endogenous opioids do not contribute significantly to JNJ63955918-induced insensitivity to pain. However, naloxone reversal experiments and more detailed dose-response studies in tolerant and non-tolerant animals are needed in order to definitively preclude a minor involvement of such a mechanism.

Our behavioral studies show that both JNJ63955918 and ProTX-II exhibit steep dose response curves for analgesic activity, suggesting that this may be a feature of Nav1.7 blockers. Indeed, although homozygous patients exhibit a painless phenotype, heterozygous Nav1.7-null subjects exhibit normal pain sensation. One possible explanation for this is that a high degree of Nav1.7 block is required for analgesia. It has long been known that a large safety factor exists for sensory transmission. Therefore, it is not surprising that significant (probably >80% based on early estimates of safety factors in peripheral nerves) block of Nav1.7 may be required for strong efficacy. The requirement for block of a significant fraction of Nav1.7 channels may also explain why the selective, closed-state blocker JNJ63955918 exhibits profound analgesia whereas moderately selective, inactivated state- and voltage-dependent small molecules have generally been unable to recapitulate the dramatic and selective analgesia observed in Nav1.7 null subjects. It seems possible that channel block by voltage-dependent small molecules may only be partial under (patho)physiological conditions and insufficient to effectively prevent transmission in nociceptive sensory nerve fibers. Indeed, non-selective state-dependent sodium channel blockers (i.e. local anesthetics) exhibit strong efficacy against acute nociceptive pain only when administered at high concentrations in the immediate vicinity of sensory nerves, concentrations that in all likelihood inhibit sodium channels in multiple states.

Our studies demonstrate that intrathecal JNJ63955918 exhibits strong efficacy at doses that do not exert any motor impairment. Further improvements in therapeutic margin will be achieved through optimization of dose volume and baricity^43^. In side-by-side comparisons, the therapeutic margin of JNJ63955018 was greater than that of ziconotide. Interestingly though, despite >100x selectivity for Nav1.7 over other neuronal sodium channel isoforms, such as Nav1.1, Nav1.2 and Nav1.6, the *in-vivo* safety window was somewhat smaller (7–16 fold based on a MTD of 5 μg/10 μl). Although not accurately defined in this study, the safety window for ProTX-II is also likely less than predicted on the basis of the *in-vitro* selectivity. The reasons for the lack of 1:1 translation between *in-vitro* selectivity and *in-vivo* safety are not clear, but may reflect difficulties in fully recapitulating physiological conditions in *in-vitro* studies, differences in safety factors for sensory versus motor neurons or involvement of other, non-sodium channels in the overall *in-vivo* profiles of the peptides employed in this study. Indeed, there is evidence that the pharmacological as well as biophysical properties of Nav channels can vary substantially depending on cell background and selectivity profiling against recombinant channels expressed in a DRG background might provide additional insight^44^,^45^.

JNJ63955018 and to a lesser extent, ProTX-II, were effective when administered peri-sciatically in rats. ProTX-II has previously been evaluated after peri-neural administration^19^. In those studies, ProTX-II was only effective after the blood-nerve barrier was disrupted by perineurial injection of hypertonic saline, suggesting that ProTX-II cannot normally cross the blood-nerve barrier to access its site of action on the peripheral nerve. The rather limited efficacy with peri-sciatic administration of ProTX-II seen in the present study lends further support to the argument that nerve penetration of ProTX-II may be inefficient. At an equivalent dose (correcting for differences in Nav1.7 potency), JNJ63955918 exerted robust efficacy following peri-sciatic administration. During the course of the perineural studies, we also observed evidence of JNJ63955918 but not ProTX-II activity in the contralateral limb following perineural injection. These findings indicate that JN63955918 may penetrate into peripheral nerves to a greater extent than ProTX-II.

Nav1.7 is expressed along the peripheral axons on sensory nerves and on pre-synaptic nerve terminals in the dorsal horn of the spinal cord^46^ and it is currently unclear whether peripheral or central block of Nav1.7 is superior from an efficacy standpoint. Our data shows that JNJ63955918 is strongly analgesic following either intra-thecal (exposure of central terminals) or peri-sciatic (exposure of peripheral axons) administration. Activity in the contralateral limb following uni-lateral peri-sciatic administration of high doses of JNJ63955918 further suggests that efficacy can be achieved via the periphery. These findings suggest that inhibition of Nav1.7 at any level of the pain pathway may be sufficient for efficacy, as previously suggested^47^. Although it is tempting to suggest that efficacy was superior following intra-thecal compared to peri-sciatic delivery, it is difficult to know whether similar levels of channel block were achieved following the two different routes of administration and detailed pharmacokinetic/pharmacodynamic studies will be required to determine which, if any, mode of delivery is superior.

We suggest that high levels of Nav1.7 inhibition may be required for efficacy and that this can be optimally achieved through potent, closed-state Nav1.7 block. Furthermore, significant selectivity (minimally > 100 x) for Nav1.7 over other sodium channels isoforms may be required for acceptable safety margins *in-vivo*. These observations highlight the challenges of Nav1.7 drug discovery and likely underlie the slow progress in translating the promise of Nav1.7 into meaningful clinical advances. As a potent, highly selective closed-state Nav1.7 blocker that exerts profound efficacy at well tolerated doses *in-vivo*, JNJ63955918 represents a significant step forward in the search for Nav1.7-based analgesics and formulation of JNJ63955918 or related peptides for intrathecal or sustained local delivery^48^ could provide a viable strategy for the treatment of certain forms of severe pain.

## Methods

The cell lines and cDNA constructs used were as follows: HEK293 cell lines: human Nav1.7 (Millipore), human Nav1.2 (Dr. H. A. Hartmann, University of Maryland Biotechnology Institute), human Nav1.4 and human Nav1.5 (Dr A. George, University of Pennsylvania). Tetracycline-inducible CHO cell lines: human Nav1.1 and human Nav1.6 (Chantest, Cleveland, Ohio, USA). Full length rat Nav channel cDNA clones (with accession number in brackets), were assembled (GeneWiz, Cambridge, MA, USA) based on published sequences and cloned into mammalian expression vectors: rat Nav1.7 (Accession # NM_133289.1), rat Nav1.6 (NM_019266), and rat Nav1.5 (NM_013125). Transient transfections were performed with Lipofectamine 2000 (Invitrogen). For electrophysiological experiments, cells were cotransfected with truncated CD4 (pMACs 4.1; Miltenyi Biotec GmbH, Bergisch Gladbach, Germany). All cell culture reagents were obtained from Invitrogen. Synthetic ProTX-II (PTX-4450-s) and ziconotide were purchased from Peptides international (Louisville, KY, USA).

### Peptide synthesis

The initial ProTX-II structure-activity relationship (SAR) was evaluated using recombinantly produced toxin variants. Recombinant peptides were generated using previously described methodology and contained an additional two residues (G,P) on the N-terminus resulting from the HRV3C digestion of the recombinant fusion protein^24^. Selected mutants were subsequently produced synthetically for detailed evaluation, using solid phase peptide synthesis. Fmoc automated solid phase synthesis of ProTX-II variants was performed on SymphonyX from Protein Technologies using a double coupling strategy employing HCTU and 2,4,6 collidine as the activator and base, respectively. Pre-loaded CLEAR resin (Peptides Int’l) or ChemMatrix (Sigma Aldrich) Wang resins were used to produce JNJ63955918 acid.

Wet synthesis resins were cleaved with 9.5 ml trifluoroacetic acid (TFA), 0.5 ml H_2_0, 0.5 ml Anisole, 0.5 ml thioanisole, 0.25 ml triisopropyl silane for 1.5–2 h at room temperature. Cleaved peptides were precipitated with 5-fold excess of diethyl ether added directly to the pre-filtered cleavage solution, isolated, and re-solubilized in TFA. Linear peptides were purified by preparative HPLC using a Phenomenex Luna C18(2), 100 Å pore size, 10 μ particle size, 250 mm × 21.2 mm column and a 15–48% linear gradient of acetonitrile with 0.05% TFA over 40 min. Molecular weights were confirmed by LC/MS and fractions were pooled for folding.

Purified linear fractions were added directly to 20 mM Tris, 2 M Urea, 1:1 oxidized/reduced glutathione, and pH was adjusted to 7.8–8.0 using acetic acid. Solutions were stirred for 24–48 h at room temperature. Folded peptides were purified using a Phenomenex Luna C18(2), 100 Å pore size, 10 μ particle size, 250 mm × 21.2 mm column with a 15–48% linear gradient of acetonitrile with 0.05% TFA over 40 min. Main peak fractions were analyzed by HPLC and LC/MS (Supplementary Figure 1). Final peptides were flash frozen and lyophilized.

Peptide yields were measured by absorbance at 280 nm using the calculated extinction coefficient. Percent purity was determined by HPLC using a Phenomenex Luna C18(2) analytical column, 250 mm × 4.6 mm, 100 Å pore size, 5 μ particle size. Peptide mass and oxidation were confirmed by LC/MS using a Waters 2965 separations module coupled to a Waters Micromass ZQ electrospray mass spectrometer.

### NMR

NMR samples were prepared by dissolving neat peptide:counter ion (trifluoroacetic acid) complex in 20 mM phosphate, 0.1 mM deuterated EDTA, and 0.002% NaN_3_ in 10% ^2^H_2_O. This gave a 1.5 mM sample in aqueous buffer at pH 6.7. All 2D NMR experiments were run at 305.56 K on a 950 MHz Bruker Avance spectrometer. The following experiments were collected: ^1^H-TOCSY^49^, ^1^H-NOESY^50^, ^15^N-HSQC^51^, ^13^C-HSQC^51^, and a ^1^H (1D) temperature series at 278, 283, 288, 293, 298, 303, 308, 313, and 318 K.

Proton spin systems for individual residues were assigned using TOCSY experiments using a spin-lock MLEV mixing times of 75 ms. Sequential residue assignments were identified using NOESY experiments collected with mixing times of 150 ms. The heteronuclear HSQC experiments helped distinguish overlapping resonances in the TOCSY and NOESY spectrums. Zero filling and shifted sinebell squared weighting was applied prior to Fourier transformation using NMRPipe for data processing^52^. Greater than 95% backbone ^1^H resonances have been assigned (H, Hα, Hβ*). Complete 100% backbone and sidechain ^15^N resonances and approximately 30% βC’s have been assigned (see Supplementary Tables 4 and 5 for chemical shift tables; BMRB accession number 30181).

CYANA v2.1 was used for initial structure generation, NOE assignment, and interproton distance restraint identification^53^. Initial ‘seed’ restraints were supplied to CYANA, specifically: 32 manually assigned NOE’s were set as distance restraints; 28 phi, psi and omega dihedral angle restraints, as predicted from chemical shifts by PREDITOR (see Supplementary Table 6 for dihedral angle ranges)^54^; and based on chemical shifts and NOEs 3 disulfide bonds were fixed between residues 2–16, 9–21, and 15–25. Amide temperature coefficients were measured, however no consecutive residues of three or more with coefficients <5 ppb/K were found therefore no manual hydrogen bond restraints were applied; see supplementary data for amide temperature coefficients. Automatic NOEs were iteratively assigned during the CYANA workflow finishing with a total of 366 unique distance restraints derived from a total of 1466 NOESY peaks. The final models used 366 CYANA determined distance restraints, 32 manually assigned distance restraints, 28 (x3) manually assigned dihedral angle restraints, and 3 manually assigned disulfide restraints to give a total of 485 restraints or approximately 16 restraints/residue. The CYANA workflow consisted of six cycles of combined automated NOE assignment and structure calculation. Each cycle calculated 1,000 conformers using a simulated annealing schedule with 10,000 torsion angle dynamics steps per conformer followed by 50,000 steps of energy minimization. The statistics for the final CYANA run are included in the supplementary information. Following initial structure generation by CYANA, MOE v2014.09 was used for restrained, explicit solvent refinement (Chemical Computing Group Inc., Montreal, Canada). The 366 distance restraints determined by CYANA were used during explicit solvent refinement in MOE.

### QPatch

Cell preparation for QPatch (QP) assays was performed as previously described^24^,^55^. Extracellular solution contained 137 mM NaCl, 5.4 mM KCl, 1 mM MgCl_2_, 2 mM CaCl_2_, 5 mM glucose, and 10 mM HEPES, pH 7.4, 315 mosM/l. In some experiments with Nav1.7, Nav1.4 and Nav1.5, extracellular Na concentration was reduced (typically by 25 or 50%) by replacement of sodium with choline to limit the amplitude of the sodium current. Intracellular solution contained 135 mM CsF, 10 mM CsCl, 5 mM EGTA, 5 mM NaCl, and 10 mM HEPES, pH 7.3, 290 mosM/l. During recordings cells were held at a holding potential equal to the V½ of steady-state inactivation for the respective channels (−75 mV for Nav1.7, Nav1.4; −65 mV for Nav1.2; −60 mV for Nav1.6 and Nav1.1 and −105 mV for Nav1.5). To elicit sodium currents cells were first hyperpolarized from the holding potential to −120 mV for 2 s and then depolarized to 0 mV for 5 ms before returning to the holding potential. This protocol was repeated once every 60 s during liquid applications. A total of five applications of the extracellular solution (1× control buffer, 3× test compound/control, 1x positive control (1 μM tetrodotoxin or 10 mM lidocaine for Nav1.5)), all containing 0.1% bovine serum albumin (BSA) except for the positive control solution) were made on each cell. The voltage protocol was executed 10 times after each application. Currents were sampled at 25 kHz and filtered at 5 kHz with an 8-pole Bessel filter. The series resistance compensation level was set at 80%. All experiments were performed at room temperature (∼22 °C). pIC_50_ (−log_10_(IC_50_)) and SEM values were determined from logistic fits of concentration-response data using GraphPad Prism.

### Manual Electrophysiology

Cells for use in manual patch-clamp (MPC) electrophysiological assays were plated at low density onto glass coverslips and maintained in appropriate media. On the day of the experiment, glass coverslips were placed in a bath on the stage of an inverted microscope and perfused (∼1 ml/min) with an extracellular solution of the following composition: 137 mM NaCl (in some experiments with Nav1.7, Nav1.4 and Nav1.5, extracellular Na concentration was reduced (typically by 50%) by replacement of choline to limit the amplitude of the sodium current), 2 mM CaCl_2_, 5.4 mM KCl, 1 mM MgCl_2_, 5 mM glucose, 10 mM HEPES, and 0.1% bovine serum albumin, pH 7.4, 315 mosM/l. Pipettes were filled with an intracellular solution of the following composition: 135 mM CsF, 10 mM CsCl, 5 mM EGTA, 5 mM NaCl, and 10 mM HEPES, pH 7.3, 290 mosM/l. All recordings were made at room temperature (22–24 °C) using a Multiclamp 700A amplifier and pClamp 9 software (Axon Instruments). Transiently transfected cells were used 48 h post-transfection and identified using anti-CD4-coated beads (Dynabeads, Invitrogen). Sodium currents were measured using the whole-cell configuration of the patch clamp technique at a test potential of −10 mV from a holding potential of −120 mV. Current records were acquired at 10 KHz and filtered at 4 KHz. Uncompensated series resistance was typically <10 megaohms, and 75% series resistance compensation was routinely applied. Currents were elicited once every 30–60 s and were allowed to stabilize for 5–10 min before recording. Compounds were applied using an SF-77B Fast-Step Perfusion device (Warner Instruments). The voltage dependence of activation was assessed with a series of test pulses to voltages in the range −80 to +40 mV from a holding potential of −120 mV. Current (I) was converted to conductance (G) by normalizing for driving force (V) according to G = I/(V_step_ − V_rev_). V_rev_ (reversal potential) for Nav1.7 was estimated to be +40 mV. The voltage dependence of inactivation was assessed using a two-pulse protocol involving a 1 s pre-pulse to voltages in the range −120 to −30 mV followed by 10 ms test step to −10 mV. Normalized inactivation and conductance curves were fit with a Boltzmann function to derive V_½_ and slope. Activation and inactivation curves under control conditions and in the presence of JNJ63955918 were constructed in different cells, 10 min after establishing whole-cell access in order to limit the confounding effects of time-dependent shifts in voltage dependent properties^56^.

### Opioid receptor studies

JNJ63955918 was evaluated for effects at human opiate-receptors using Cisbio HTRF Dynamic Range cAMP detection kits (Cisbio#62AM4PEC) and assay protocols, with human opioid receptor cell lines provided by Chantest (Mu: ChanTest #CT6605 or #CT4605, Genbank Accession # NM_000914; Kappa: ChanTest #CT6606, Genbank Accession # NM_000912; Delta: ChanTest #CT6607, Genbank Accession # NM_000911.2).

### Animals

All procedures and experiments involving animals were carried out in accordance with internationally accepted guidelines for the care and use of laboratory animals in research and all animal protocols were approved by the Institutional Animal Care and Use Committee of Johnson & Johnson Pharmaceutical Research & Development, L.L.C. or the University of California, San Diego. Male Sprague-Dawley (CD) rats, 250~300 grams (Charles River, San Diego) were housed in cages with corncob bedding, with *ad libitum* access to chow and water.

### Rat Dorsal Root Ganglion studies

L4-L6 dorsal root ganglia (DRG) were aseptically excised, washed in ice-cold Tyrode’s solution (145 mM NaCl, 4 mM KCl, 1 mM CaCl_2_, 1 mM MgCl_2_, 10 mM dextrose, and 10 mM HEPES, pH 7.3), and incubated in Tyrode’s solution containing 2 mg/ml collagenase and 1 mg/ml protease for 45 min at 37 °C with 5% CO_2_. After digestion, ganglia were washed in Tyrode’s solution, transferred into 1 ml of Dulbecco’s modified Eagle’s medium with 10% fetal bovine serum, and single cells were liberated by gentle mechanical agitation using a disposable transfer pipette. Cells were plated on poly-D-lysine coated coverslips, and maintained at 37 °C. 3–36 h post-isolation, cover slips were placed in a bath on the stage of an inverted microscope and perfused (approximately 1 ml/min) with extracellular solution. Solutions used for recording TTXs and TTXr currents were as follows: Extracellular solution 41 mM NaCl, 96 mM choline Cl, 2 mM CaCl_2_, 4 mM KCl, 1 mM MgCl_2_, 5 mM glucose, 10 mM HEPES, 14 mM TEA Cl, 0.2 mM CdCl_2_, 0.1% BSA, pH 7.4, 315 mosM/l. Intracellular solution: 135 mM CsF, 10 mM CsCl, 5 mM EGTA, 5 mM NaCl, and 10 mM HEPES, pH 7.3, 290 mosM/l. Solutions for recording persistent currents were as follows: Extracellular solution: 68.5 mM NaCl, 68.5 mM choline Cl, 2 mM CaCl_2_, 4 mM KCl, 1 mM MgCl_2_, 5 mM glucose, 10 mM HEPES, 10 mM TEA Cl, 0.2 mM CdCl_2_, 0.0005mM TTX, 0.1% BSA, pH 7.4, 315 mosM/l. Intracellular solution: 30 mM CsF, 100 mM CsCl, 10 mM EGTA, 1 mM CaCl_2_, 8 mM NaCl, 1 mM MgCl_2_, 10 mM HEPES, 0.4 mM Na_2_GTP and 4 mM MgATP, pH 7.3, 292 mosM/l^57^. Pipettes were filled with an intracellular solution as described above, and had a resistance of 1 to 2 MΩ. All recordings were made at room temperature (22–24 °C) using a Multiclamp 700A amplifier and pClamp 10 software (Axon Instruments). Current records were acquired at 20 kHz and filtered at 4 kHz. Drugs were applied using an SF-77B Fast-Step Perfusion device (Warner Instruments). TTXs and TTXr sodium currents were elicited by a 20 ms step to 0 mV after a brief (100 ms) step to −120 mV from a holding potential of −80 mV, once every 20 s. The amplitude of the TTX-sensitive current was determined by digital subtraction of the current elicited in the presence of 1 μM TTX. Persistent currents were elicited by a 100 ms step to −55 mV or −50 mV from a holding potential of −100 mV, once every 20 s. The amplitude of the persistent sodium current was determined by digital subtraction of the current elicited in the absence of extracellular sodium.

### Rat IT studies

Rats were placed in a stereotaxic head holder under gaseous anesthesia (4% isoflurane for induction and 2% for maintenance). The hair over the occipital area was shaved and the area scrubbed. A 1 cm, dorsal midline incision was made to expose the atlanto-occipital membrane. A sterilized catheter (Polyethylene, PE8: 0.008” × 0.014” OD) was inserted through a 2 mm incision into the intrathecal space and advanced caudally to the level of the L1–L2 spinal segments. The external end of the catheter (PE10) was tunneled through the skin and occluded with a 28-gauge stainless steel wire. The incision site was closed with sterile, non-absorbable, monofilament 4–0 nylon suture and wound clips. Intrathecally-cannulated animals were housed individually and allowed to recover for at least 5 d before the behavior experiments were performed. Rats were monitored daily and removed from the study if any neurological dysfunction was noted, if there was >15% weight loss over 5 d, or if the catheters were occluded.

### Test Compound Delivery

All peptides were formulated in DPBS (no Ca^2+^ and Mg^2+^) and stored at 4 °C until use. For bolus administration, 10 μl of compound solution or vehicle were injected via the IT catheter followed by 10 μl saline flush. For 14 d infusion, the non-indwelling end (PE60) of the IT catheter was connected to the flow modulator of an osmotic pump (Alzet 2002, 0.5 μl/h) loaded with JNJ63955918 or saline vehicle, which underwent priming at 37 °C overnight in sterile saline. The osmotic pump was implanted subcutaneously above the scapula. Animals were excluded from behavioral studies if any motor dysfunction was noted following IT injection, if baseline thresholds were >90% or <10% of the cut-off value in the tail flick, hotplate and Hargreaves test, if there was significant leak (>20 μl) while injecting formalin into the paw or any malfunction of the metal bands or formalin apparatus. Intrathecal compound was administered 15 min–1 h before commencing behavioral testing. Intrathecal-cannulated rats were trained on the behavioral apparatus for at least two days before baseline withdrawal latencies were taken. All behavioral tests were conducted by an investigator blinded to group and treatment. After compound administration, however, some rats treated with high doses of ProTX-II or JNJ63955918 developed skin abrasions/lesions. At that point blinding was no longer possible.

### Hargreaves Test

A modified Hargreaves box was used to measure thermal paw withdrawal latency (PWL)^58^,^59^. This system (University Anesthesia Research and Development Group, Department of Anesthesiology, University of California, San Diego) consisted of 6 individual chambers with a glass floor maintained at a constant temperature (27 °C). The thermal nociceptive stimulus originated from a light beam focused on the undersurface of the rat hind paw. The intensity of the stimulus was adjusted by the delivered amperage to induce paw withdrawal latency (PWL) of 8–12 s in normal animals. A 20 s cutoff time was used to prevent possible tissue damage. Animals were allowed to habituate on the glass surface for 30–60 min before PWL measurement. Three PWL measurements, at least 5 min apart, were made and averaged for each hind paw.

### Hotplate test

Animals were placed on a 10″ × 10″ metal plate surrounded by 4 Plexiglas walls (15″ high). The plate was maintained at a temperature of 52.5 °C. The response latency (time when the animal first flinches or licks its hind paw, jumps, or vocalizes) was measured and the animal removed from the plate. Animals showing no response were removed from the plate after 30 s to prevent any possible tissue damage.

### Tail-flick test

Animals were placed on a tail-flick device (Ugo Basile). The device has a focal infrared light heating area (diameter-5 mm). The tail (1/3–1/2 way from distal end) was placed in the focal heating area. The temperature of the heat source was adjusted to elicit a tail-flick within 10 s in vehicle treated animals. A 15 s cut-off time was used to prevent tissue damage. The time elapsed between the start of the heat stimulus and any tail movement is measured automatically.

### Formalin Flinching

A soft metal band was glued to the plantar side of one hind paw and rats were allowed to acclimate in the test chamber for 30 min. The dorsum of the banded paw was injected with 50 μl of 2.5% formalin and the animals were immediately placed back into the test chamber. The incidence of nociceptive behavior - flinching and shaking of the injected paw was counted by an automated formalin apparatus (University Anesthesia Research and Development Group, Department of Anesthesiology, University of California, San Diego, La Jolla, CA). The number of paw flinches was tallied by minute over 60 min^60^. Total flinches in phase I (0~10 min) and phase II (11~60 min) were calculated.

### Morphine tolerance

Male Sprague-Dawley (CD) rats (Charles River, San Diego) ~250–300 g were catheterized intrathecally as described above. The non-indwelling end (PE60) of the catheter was connected to the flow modulator of an osmotic pump (Alzet 2001, 1 μl/h) loaded with morphine or saline vehicle, which underwent priming at 37 °C overnight in sterile saline. The osmotic pump was implanted subcutaneously above the scapula. To induce morphine tolerance, morphine was infused intrathecally by the osmotic pump at a dose of 15 μg/h for 7 days^61^. On day 7 the pumps were extracted and the wounds were closed with the intrathecal catheters left in place. Catheters were flushed with 20 μL of saline. Rats were allowed to recover for approximately 3 h before receiving intrathecal injection of PBS, JNJ63955918 or morphine. The formalin test was performed 1 h following JNJ63955918 or 15 min following morphine injection.

### Rat Perineural studies

Rats ~250–300 g were placed under Isoflurane anesthesia (4% for induction, 2% for maintenance). A single hip and proximal hind-leg area were shaved and the injection area prepped with alternating applications of chlorhexidine 2% soln. and isopropyl alcohol 70%. A pelvic notch just distal to the greater trochanter was located with palpation and a starter hole was made through the dermis using a 20G hypodermic needle. A 22G nerve stimulating injection needle (ProBloc II, cat#HN3S-40, Halyard Health) connected to a peripheral nerve stimulation device (EZstim II model ES400, Life-Tech, Inc.) was placed into the starter hole. The nerve stimulator was set to 0.7 mA and the injection needle was gently advanced in a line parallel to the femur until localized dorsal or plantar flexion of the foot was observed. Generalized muscle twitching involving primary twitching on the quadriceps muscle was considered a negative response and the needle was carefully re-positioned until the appropriate localized foot flicking was observed. Upon observation of the localized foot twitching, the amperage was slowly reduced to 0.2 mA. If the foot twitching was observed at 0.2 mA the device was lowered to 0.1 mA, where, if the needle was appropriately placed, the twitching ceased. The device was increased back to 0.2 mA and if twitching was still observed, the 100 μL injection was given. If the twitching stopped before reaching the 0.2 mA threshold, or if the twitching was observed at 0.1 mA, the needle was adjusted accordingly. Upon successful injection the animals were recovered and their thermal latencies were assessed at 10, 15, 30, 60, 120, 240 min, and 24 h post injection for ProTX-II or 15, 30, 60, 120, 240 min, and 24 h post injection for JNJ63955918.

### Data Analysis

Behavioral data are represented as mean ± s.e.m. Statistical analysis was performed using Prism (Graphpad Software Inc., La Jolla, CA). Data was analyzed using ANOVA with Bonferroni post-hoc test (one- or two way, as indicated in the results). Four parameter logistic regression was used to calculate ED_50_ values. For hotplate and tail flick data, the area under the curve (%AUC) for the first 120min after compound administration was used to calculate ED_50_ values. First, %MPE (percent maximum possible effect) was calculated for each time point after compound administration. %MPE = (latency − baseline latency)/(cut-off latency − baseline latency) × 100%. The %AUC was calculated using the %MPE values by the trapezoidal rule. The maximum %AUC equals 100, which means an animal’s latency reaches cut-off value at all time points. To calculate the ED_50_ of JNJ63955918 against the formalin phase II response, number of flinches was converted into %inhibition for each rat. %Inhibition = (1− number of flinches/mean number of flinches of the control group) × 100.

## Additional Information

**How to cite this article**: Flinspach, M. *et al*. Insensitivity to pain induced by a potent selective closed-state Nav1.7 inhibitor. *Sci. Rep.* **7**, 39662; doi: 10.1038/srep39662 (2017).

**Publisher's note:** Springer Nature remains neutral with regard to jurisdictional claims in published maps and institutional affiliations.

## Supplementary Material

Supplementary Information

## Figures and Tables

**Table 1:** Potency and selectivity of synthetic ProTX-II and JNJ63955918.

Peptide		hNav1.7	hNav1.1	hNav1.2	hNav1.3	hNav1.4	hNav1.5	hNav1.6
ProTX-II QP	pIC_50_	9.1 ± 0.06	7.8 ± 0.08	7.1 ± 0.07	7.6 ± 0.09	7.1 ± 0.07	6.4 ± 0.07	7.5 ± 0.04
	Fitted E_max_ (%)	88.8 ± 3.1	100	100	100	100	100	100
	Fold Nav1.7 Selectivity	—	19.8	99.3	31.4	99.3	497.6	39.5
JNJ63955918 QP	pIC_50_	8.0 ± 0.09	5.2 ± 0.13	6.1 ± 0.38	nd	4.8 ± 0.13	<5.5	5.6 ± 0.11
	Fitted E_max_ (%)	79.1 ± 3.4	100	27.6 ± 7.3	nd	100		100
	Fold Nav1.7 Selectivity	—	631	79.4	nd	1584.9	>316.2	251.2
JNJ63955918 MPC	pIC_50_	8.0 ± 0.08	5.0 ± 0.06	5.8 ± 0.46	nd	5.3 ± 0.11	<5.0	6.0 ± 0.04
	Fitted Emax (%)	94.8 ± 3.3	100	50.0 ± 18.5	nd	100		100
	Fold Nav1.7 Selectivity	—	1000	158.5	nd	501.2	>1000	100

(IC_50_ is defined as the concentration to produce inhibition equivalent to 50% of the fitted E_max_).

QP = IC_50_ determined by automated electrophysiology using QPatch.

MPC = IC_50_ determined by conventional manual patch-clamp electrophysiology.

Fold Nav1.7 selectivity = 10^(Nav1.7 pIC_50_ − Nav1.x pIC_50_).

**Table 2:** Summary of *in-vivo* pharmacology of JNJ63955918.

Model	Compound	Route	pED_50_ (−log g)
Hotplate	JNJ63955918	i.t. bolus	6.1 ± 0.06
Tail Flick	JNJ63955918	i.t. bolus	6.5 ± 0.06
Formalin (phase II)	JNJ63955918	i.t. bolus	6.3 ± 0.15
Formalin (phase II)	ProTX-II	i.t. bolus	>5.7
Formalin (phase II)	ziconotide	i.t. bolus	7.1 ± 0.18
Formalin (phase II)	morphine	i.t. bolus	5.4 ± 0.22
