# Supplementary material for: Insensitivity to pain induced by a potent selective closed-state Nav1.7 inhibitor
Source: Sci Rep. 2017 Jan 3;7:39662. doi: 10.1038/srep39662 (PMC5206724; doi:10.1038/srep39662)
Supplement: Supplementary Information [file srep39662-s1.pdf]

Supplementary Information

Insensitivity to pain induced by a potent selective closed-state Nav1.7 inhibitor

[1]Flinspach, M., [1#]Xu, Q., [1#]Piekarz, A.D., [1#]Fellows, R., [1]Hagan, R., [1]Gibbs, A., [1]Liu, Y., [1]Neff, R.A., [1]Freedman, J., [1]Eckert, W.A. 3rd, [1]Zhou, M., [1]Bonesteel, R., [2]Pennington, M.W., [3]Eddinger, K.A., [3]Yaksh, T.L., [1]Hunter, M., [1]Swanson, R.V., and [*1]Wickenden, A.D.

[1]Janssen R&D, L.L.C., 3210 Merryfield Row, San Diego, CA 92121, [2]Peptides International, Louisville, KY 40299 and [3]University of California, San Diego, Department Anesthesiology and Pharmacology, 9500 Gilman Drive, La Jolla, CA 92093-0818

Supplementary Figure 1: MS and HPLC characterization of JNJ63955918 batches

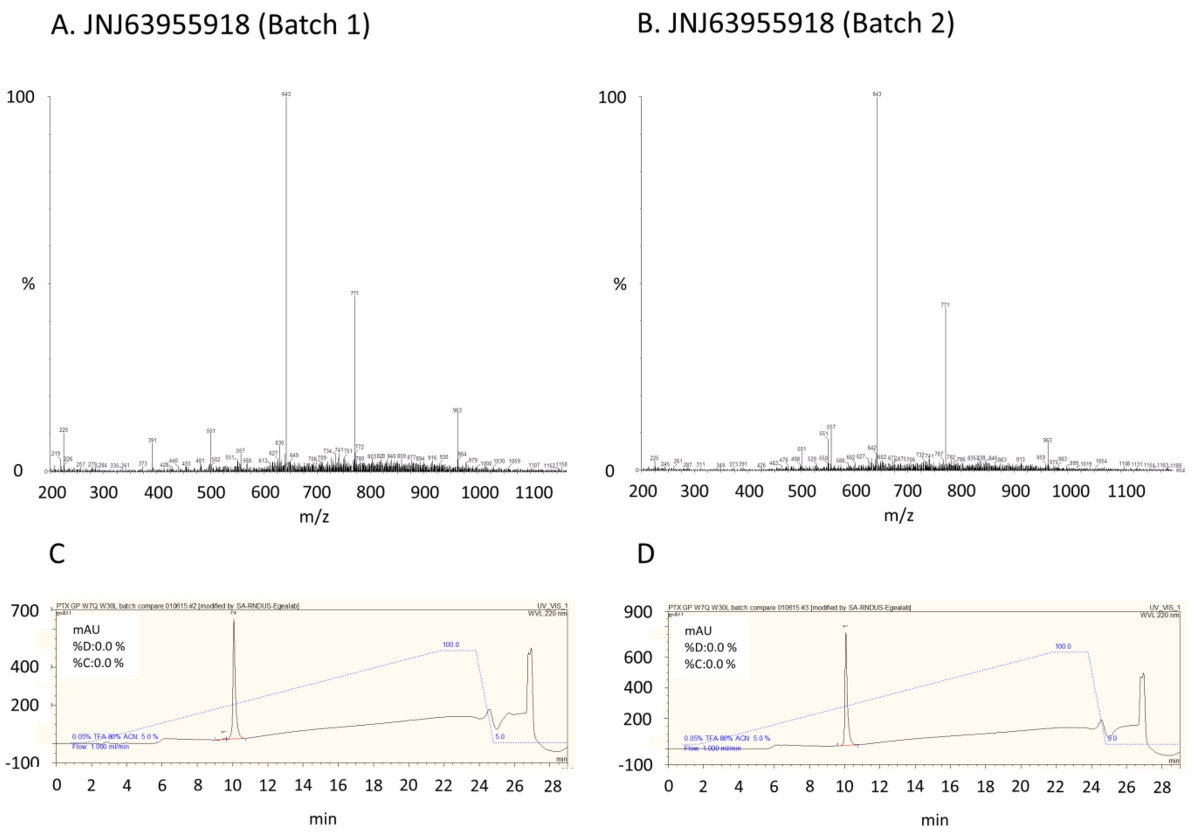

Supplementary Figure 2. Side by side comparison of the two structure ensembles of protoxin-II (2N9T) and JNJ63955918. The global ICK fold is clearly evident between the two highly homologous peptides. 20 lowest energy conformers for each ensemble are shown.

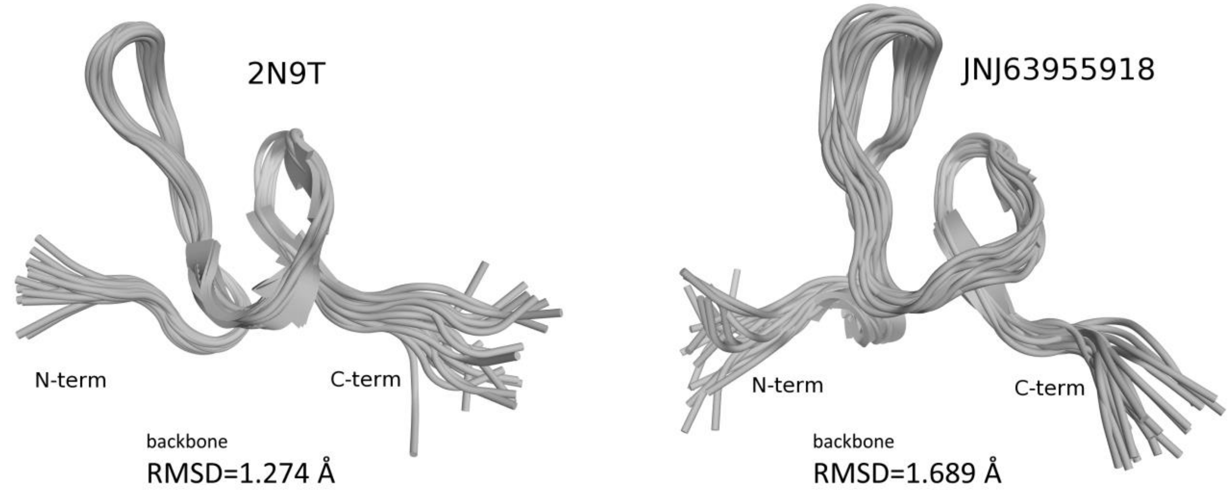

Supplementary Figure 3. Effect of continuous intrathecal infusion of either vehicle (black circles) or 0.5 μg/h JNJ63955918 on rat body weight (red circles).

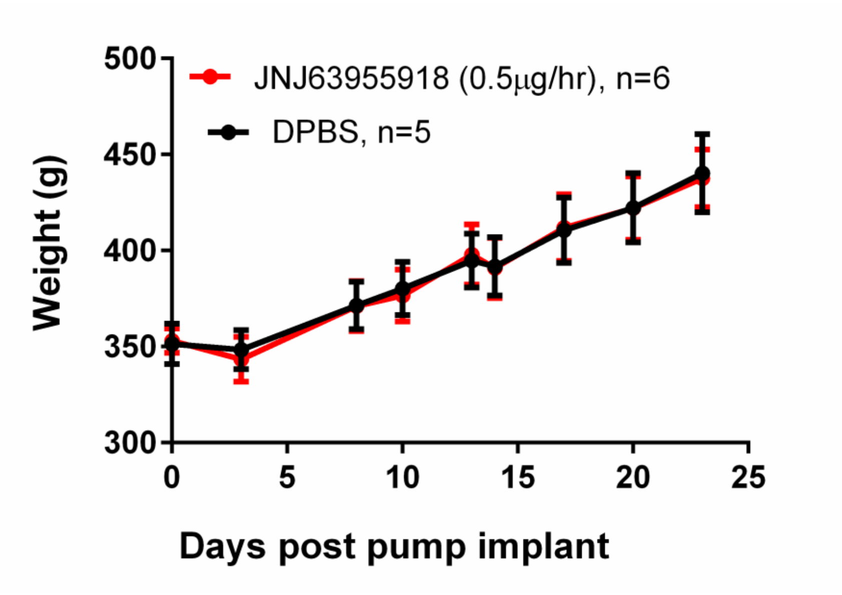

Supplementary Table 1: Gross behavioral assessments during rat formalin assay (number of observations/total number of animals)

	2 μg/10 μl ProTX-II			vehicle		
	Baseline	30 min post IT dosing	90 min post IT dosing	Baseline	30 min post IT dosing	90 min post IT dosing
Hindlimb weakness	0/7	2/7	2/7	0/7	0/7	0/7
Flaccidity	0/7	1/7	0/7	0/7	0/7	0/7
Abnormal stepping	0/7	2/7	0/7	0/7	0/7	0/7
Abnormal proprioception of hind paws	0/7	2/7	0/7	0/7	0/7	0/7

Supplementary Table 2: Gross behavioral observations in animals dosed intrathecally with either JNJ63955918 or vehicle (number of observations/total number of animals)

	Baseline	1 h post dose	2 h post dose (1 h post formalin)
2.5 μg/10 μl JNJ63955918, IT			
Normal proprioception	6/6	6/6	6/6
Gross motor normal	6/6	6/6	6/6
Scatching	2/4*		
Abrasions at euthanasia	2/4*		
0.5 μg/10 μl JNJ63655918, IT			
Normal proprioception	6/6	6/6	6/6
Gross motor normal	6/6	6/6	6/6
Scatching	1/4*		
Abrasions at euthanasia	0/4*		
10 μl vehicle, IT			
Normal proprioception	6/6	6/6	6/6
Gross motor normal	6/6	6/6	6/6
Scatching	0/4*		
Abrasions at euthanasia	0/4*		

*The first 2 animals dosed were not assessed for scratching behavior

Supplementary Table 3: Gross behavioral observations in animals dosed perineurally with either JNJ63955918 or vehicle (number of observations/total number of animals)

1.4 mg/100 μl JNJ63955918, perineural							
	Baseline	15 min post dose	30 min post dose	60 min post dose	120 min post dose	240 min post dose	24 h post dose
Mentation BAR	9/9	7/9	4/9	5/9	6/9	8/9	9/9
Scratching	0/9	0/9	1/9	2/9	2/9	1/9	0/0
Normal placing stepping	9/9	9/9	9/9	9/9	9/9	9/9	9/9
Normal pinna reflex	9/9	9/9	9/9	9/9	9/9	9/9	9/9
Normal corneal reflex	9/9	9/9	9/9	9/9	9/9	9/9	9/9
100 μl vehicle, perineural							
Mentation BAR	9/9	2/9	5/9	4/9	8/9	8/9	9/9
Scratching	0/0	0/9	0/9	0/9	0/9	0/9	0/9
Normal placing stepping	9/9	9/9	9/9	9/9	9/9	9/9	9/9
Normal pinna reflex	9/9	9/9	9/9	9/9	9/9	9/9	9/9
Normal corneal reflex	9/9	9/9	9/9	9/9	9/9	9/9	9/9

BAR = Bright, alert and responsive

Supplementary Table 4. Chemical Shifts

Protoxin-II

	HN	HA	HB	Other
Y1		4.2115	3.161	HD1_2 7.2031, HE1_2 6.9570
C2		5.0609	3.1164	2.915 (Cb43.2163)
Q3	9.214	4.4617	2.7309	2.3293 2.1668 2.0517
K4	5.1822	3.683	2.8269	1.4203 1.3338 1.2939 0.6421 0.3961
W5	7.9478	4.2985	3.0930	(Cb28.3347) HE1_10.810, 7.1576, HZ2_7.5441, HD1_7.4554, 7.2546
M6	9.1106	3.8933	1.9078/1.6704	g(1.2927/0.5625)
W7	8.6772	5.03	3.6056	3.4401 (Cb29.7581) HE1_10.231, 7.7260, HZ2_7.6103, 7.3394, HD1_7.0922
T8	8.5078	4.548	4.289	1.2893
C9	7.8131	5.1643	3.4185	3.3106 (Cb48.1337)
D10	8.7346	4.4038	3.1596	
S11	8.3634	4.089	3.9739	3.9115 (cb62.8860)
E12	8.3069	4.4185	2.2387	2.1812 2.1164 1.9797
R13	8.2638	4.512	3.1991	3.038 1.8071 1.6200 1.4114
K14	7.9251	4.3969	1.7568	1.4618
C15	9.1623	4.9077	2.9797	2.5265 (Cb40.6282)
C16	9.0877	4.5192	3.3546	2.3682 (Cb40.0497)
E17	8.3557	4.0444	2.2918	2.2755 2.0017 1.9526
G18	8.8465	4.2621	3.6815	(Ca44.9684)
M19	7.5176	5.0639	2.3538	2.1452
V20	9.0762	4.4761	1.9078	0.8287
C21	8.9355	4.7782	3.3106	2.8358 (Cb39.7224)
R22	8.2236	4.512	3.2499	3.0875 1.6848 1.5481 1.4402
L23	8.0836	3.7221	2.1543	1.3842 1.0611 0.8273 0.786
W24	8.0715	5.3034	2.9941	2.6919 (Cb32.0874)HE1_10.245, HD1_7.0335, 7.4989,HZ2_7.4480, 7.2166, 7.1514
C25	8.6399	4.8861	3.3682	3.1452 (Cb40.1106)
K26	9.2915	4.9725	2.7873	2.0445 1.6992 1.5625 1.4762
K27	8.5509	4.2531	1.7351	1.6056 1.2946 1.1967 1.1268
K28	8.0887	4.066	2.6975	1.6200 1.4474 1.1161 1.0679
L29	8.0571	4.2674	1.4546	0.8699 0.7720 (Cb42.3041)
W30	7.3998	4.4545	3.3322	3.1811 (Cb29.8875) HE1_10.098, 7.6374,HZ2_7.4748, 7.2069, 7.1499, HD1_7.1776

JNJ63955918

	HN	HA	HB	Other
Y3	8.2849	4.6459	3.0551	2.9383 (Cb38.4484) (74, 75) (aryl 7.1011)
C4	7.7968	4.8998	3.0733	2.8776 (Cb43.1327)
Q5	9.1551	4.3423	2.5708	1.9525 1.8161
K6	8.4224	4.1170	2.0355	1.6856 1.6382 1.5730 1.5255 1.3951
W7	8.5032	4.7515	3.1622	2.8717 (Cb38.448) HE1 10.7031 7.4091 7.1354
M8	9.1088	3.7671	1.6738	1.5908 1.2468 0.4640 (noe C25)
Q9	8.2893	4.5442(4.0100)		2.3351 2.2049 2.0861 (sidechain NH 7.5268/6.8162)
T10	8.3227	4.4048	4.1977	1.2053
C11	7.6877	5.0599	3.3105	3.1385 (Cb48.2416)
D12	8.6342	4.3246	3.0733	
S13	8.2736	4.0100	3.8855	3.8346 (Cb62.8701)
E14	8.2223	4.3542	2.1897	2.1304 2.0355 1.9051
R15	8.1609	4.4313	3.1089	2.9547 1.6738 1.4840 1.2883
K16	7.8104	4.3008	1.8220	1.6679 1.6086 1.3476
C17	9.0387	4.8670	2.9395	2.4637 (Cb40.6832) (63, 64)
C18	9.3761	4.5084	3.3698	2.4269 (Cb40.0179) (66, 69)
E19	7.9441	4.9709	2.9132	2.5514
G20	8.8060	4.2593	3.6485	(Ca 44.8998)
M21	7.4922	5.0540	2.3320	2.1185 1.8635
V22	9.0493	4.4032	1.8433	0.7590
C23	8.8605	4.6804	3.2334	2.7531 (Cb39.8496) (72, 73)
R24	8.1177	4.4491	3.1978	3.0377 1.6086 1.4662 1.3595
L25	7.9964	3.6189	2.0355	1.2883 0.9503/0.8969
W26	7.9713	5.1548	2.8420	2.5514 (Cb31.9221) HE1 10.1412 7.3885 6.9121
C27	8.4467	4.8227	3.2216	3.1148 (Cb39.8496)
K28	9.2369	4.9176	1.9584	1.6323 1.4840 1.3891
K29	8.4575	4.2475	1.7331	1.5730 1.5196 1.2883 1.1934 1.0986
K30	8.0692	4.1289	1.6916	1.5137 1.2527
L31	8.2577	4.2949	1.5409	0.8667 0.7759
L32	7.6631	4.1170	1.5018	0.8182

Supplementary Table 5. JNJ63955918 Structure Statistics

NMR distance & dihedral constraints	
Distance constraints	
Total NOE	395
Intra-residue	118
Inter-residue	277
Sequential (|i-j| < = 1)	241
Medium-range (|i-j| < 5)	47
Long-range (|i-j > 5)	107
Intermolecular	NA
Hydrogen bonds	NA
Total dihedral angle restraints	
Phi	28
Psi	28
Structure Statistics	
Violations (mean and s.d.)	
Distance constraints (Å)	0.205854 (0.111399)
Dihedral angle constraints (°)	11.2517 (3.25571)
Max. dihedral angle violation (°)	21.92
Max. distance constraint violation (Å)	0.77
Deviations from idealized geometry	
Bond lengths (Å)	0
Bond angles (°)	pCA-pC-N:116.70 +/- 1.355
	pC-N-CA: 121.60 +/- 1.554
	N-CA-C: 110.80 +/- 2.506
	CA-C=O: 120.50 +/- 1.055
	N-CA-CB: 110.60 +/- 1.420
Average pairwise r.m.s.d.** (Å)	
Heavy	1.20 +/- 0.13
Backbone	0.79 +/- 0.15

Supplementary Table 6: Dihedral angle restraints used in structure calculations

3	TYR	PHI	-40.00	-120.00
4	CYSS	PHI	-30.00	-110.00
5	GLN	PHI	-40.00	-90.00
6	LYS	PHI	-40.00	-90.00
7	TRP	PHI	-40.00	-90.00
8	MET	PHI	-40.00	-90.00
9	GLN	PHI	-40.00	-90.00
10	THR	PHI	-40.00	-90.00
11	CYSS	PHI	-40.00	-90.00
12	ASP	PHI	-40.00	-90.00
13	SER	PHI	-40.00	-90.00
14	GLU	PHI	-40.00	-100.00
15	ARG	PHI	-40.00	-90.00
16	LYS	PHI	-40.00	-100.00
17	CYSS	PHI	-30.00	-110.00
18	CYSS	PHI	-40.00	-90.00
19	GLU	PHI	-40.00	-120.00
20	GLY	PHI	110.00	40.00
21	MET	PHI	-50.00	-180.00
22	VAL	PHI	-30.00	-130.00
23	CYSS	PHI	-30.00	-100.00
24	ARG	PHI	-40.00	-90.00
25	LEU	PHI	-40.00	-90.00
26	TRP	PHI	-40.00	-130.00
27	CYSS	PHI	-40.00	-90.00
28	LYS	PHI	-40.00	-90.00
29	LYS	PHI	-40.00	-90.00
30	LYS	PHI	-40.00	-150.00
3	TYR	PSI	-170.00	110.00
4	CYSS	PSI	20.00	-60.00
5	GLN	PSI	0.00	-60.00
6	LYS	PSI	-20.00	-70.00
7	TRP	PSI	-10.00	-70.00
8	MET	PSI	-10.00	-70.00
9	GLN	PSI	-10.00	-70.00
10	THR	PSI	-10.00	-70.00
11	CYSS	PSI	-10.00	-70.00
12	ASP	PSI	-10.00	-70.00
13	SER	PSI	0.00	-70.00
14	GLU	PSI	0.00	-70.00
15	ARG	PSI	-10.00	-70.00
16	LYS	PSI	10.00	-50.00
17	CYSS	PSI	10.00	-70.00
18	CYSS	PSI	10.00	-50.00
19	GLU	PSI	179.99	110.00
20	GLY	PSI	50.00	-30.00
21	MET	PSI	179.99	100.00
22	VAL	PSI	40.00	-70.00
23	CYSS	PSI	0.00	-70.00
24	ARG	PSI	10.00	-70.00
25	LEU	PSI	0.00	-70.00
26	TRP	PSI	30.00	-50.00
27	CYSS	PSI	0.00	-70.00
28	LYS	PSI	-10.00	-70.00
29	LYS	PSI	-10.00	-70.00
30	LYS	PSI	179.99	90.00
3	TYR	OMEGA	179.00	180.00
4	CYSS	OMEGA	179.00	180.00
5	GLN	OMEGA	179.00	180.00
6	LYS	OMEGA	179.00	180.00
7	TRP	OMEGA	179.00	180.00
8	MET	OMEGA	179.00	180.00
9	GLN	OMEGA	179.00	180.00
10	THR	OMEGA	179.00	180.00
11	CYSS	OMEGA	179.00	180.00
12	ASP	OMEGA	179.00	180.00
13	SER	OMEGA	179.00	180.00
14	GLU	OMEGA	179.00	180.00

15	ARG	OMEGA	179.00	180.00
16	LYS	OMEGA	179.00	180.00
17	CYSS	OMEGA	179.00	180.00
18	CYSS	OMEGA	179.00	180.00
19	GLU	OMEGA	179.00	180.00
20	GLY	OMEGA	179.00	180.00
21	MET	OMEGA	179.00	180.00
22	VAL	OMEGA	179.00	180.00
23	CYSS	OMEGA	179.00	180.00
24	ARG	OMEGA	179.00	180.00
25	LEU	OMEGA	179.00	180.00
26	TRP	OMEGA	179.00	180.00
27	CYSS	OMEGA	179.00	180.00
28	LYS	OMEGA	179.00	180.00
29	LYS	OMEGA	179.00	180.00
30	LYS	OMEGA	179.00	180.00
